# Pothole Detection System Using a Black-box Camera

**DOI:** 10.3390/s151129316

**Published:** 2015-11-19

**Authors:** Youngtae Jo, Seungki Ryu

**Affiliations:** Highway Research Institute, Korea Institute of Civil Engineering and Building Technology, Goyang-si, Gyeonggi-do 10223, Korea; E-Mail: ytjoe@kict.re.kr

**Keywords:** pothole, black-box camera, pothole detection, low cost

## Abstract

Aging roads and poor road-maintenance systems result a large number of potholes, whose numbers increase over time. Potholes jeopardize road safety and transportation efficiency. Moreover, they are often a contributing factor to car accidents. To address the problems associated with potholes, the locations and size of potholes must be determined quickly. Sophisticated road-maintenance strategies can be developed using a pothole database, which requires a specific pothole-detection system that can collect pothole information at low cost and over a wide area. However, pothole repair has long relied on manual detection efforts. Recent automatic detection systems, such as those based on vibrations or laser scanning, are insufficient to detect potholes correctly and inexpensively owing to the unstable detection of vibration-based methods and high costs of laser scanning-based methods. Thus, in this paper, we introduce a new pothole-detection system using a commercial black-box camera. The proposed system detects potholes over a wide area and at low cost. We have developed a novel pothole-detection algorithm specifically designed to work with the embedded computing environments of black-box cameras. Experimental results are presented with our proposed system, showing that potholes can be detected accurately in real-time.

## 1. Introduction

Potholes refer to any type of road surface distress on an asphalt pavement that is more than 150 mm in diameter [1]. Potholes are induced by the combined presence of water in the asphalt soil structure and heavy traffic. Potholes are mostly generated in winter and spring, because water often penetrates the pavement during these seasons. In Korea, the number of potholes was estimated at approximately 90,000 and 180,000 in 2008 and 2013, respectively. The cumulative number of potholes between 2008 and 2013 was approximately 930,000. Moreover, the number of car accidents caused by potholes between 2008 and 2013 was tallied at 4223. 

Potholes can seriously compromise driver safety and road efficiency. Many researchers and transportation experts have attempted to develop appropriate pothole-maintenance systems. In Korea, Seoul’s metropolitan government developed a pothole-maintenance system using an accelerometer and a GPS sensor equipped on a public bus [2]. The system not only warns drivers of potholes, but also alerts the relevant officials who can dispatch a road-repair crew to patch the hole within 24 h of receiving a report. 

However, most road-maintenance agencies in Korea continue to collect pothole information manually. Manual methods require considerable time and expense. Recently, automatic pothole-detection systems have been proposed, including methods that detect potholes through vibrations [3,4,5,6,7,8], methods based on laser scanning [9,10], and vision-based methods [11,12,13]. Vibration-based methods detect potholes at low cost and with simple algorithms. With these systems, however, the area wherein detections can be made is limited to the paths of vehicle wheels. Laser-scanning methods can collect extremely detailed road-surface information, but the cost of the required equipment remains significantly high. 

Rapid data collection of pothole information at low cost over a wide area is crucial for developing an efficient pothole-maintenance strategy. In other words, a specifically designed system that can collect pothole information at high speeds over a wide area is needed. Existing manual methods, along with vibration-based and laser-scanning methods are insufficient.

The goal of our research is to develop a pothole detector using common devices that are used by many drivers over a wide area. Moreover, the devices should provide high detection accuracy at low cost. We surveyed various devices, and we concluded that a black-box camera is the most suitable device for our requirement. If our proposed pothole detection algorithm is installed on a commercial black-box camera, all the vehicles on roads will be a pothole detector. As a result, we can collect huge amounts of pothole data at high speeds over a wide area. 

As mentioned earlier, vision-based methods have the potential for application as a suitable pothole detection system. Video data can be obtained at low cost using commercial cameras. Moreover, cameras can scan the road’s surface, covering a wide area. Recently, the resolution of black-box cameras has become sufficiently high for capturing details of a road’s surface. However, detecting potholes using video data has not been developed in the automotive industry, and most proposed models were implemented on desktop computers. Real-time pothole-detection systems using black-box cameras have yet to be tested in the field.

In this paper, we propose a novel pothole-detection system using a commercial black-box camera. The proposed system is mounted on the front windshield of a vehicle and can detect a pothole in real-time. A pothole-detection algorithm is installed on an embedded board in the black-box camera. This algorithm collects information regarding the size of potholes and their location, and this information is stored in the black box and then transmitted to a pothole-management server. 

The proposed pothole-detection algorithm is uniquely designed in consideration of the embedded boards in black-box cameras. We tested our algorithm with PC-based simulation software before installing it in a black-box camera. Experimental results confirmed that our algorithm performs sufficiently well for pothole detection.

The remainder of this paper is organized as follows: in the next section, we review the related literature on pothole detection. In Section 3, we describe our proposed pothole-maintenance system, and, in Section 4, we explain the proposed pothole-detection algorithm. Section 5 describes the performance evaluation. Finally, our conclusions and future work regarding this study are discussed in Section 6. 

## 2. Related Work

Recently, automatic pothole-detection systems using various sensors have been studied. Existing proposals can be categorized into vibration-based methods [3,4,5,6,7,8], laser-scanning methods [9,10], and vision-based methods [11,12,13]. 

Vibration-based methods generally use gradient variation from accelerometer data. Accelerometers have been employed for pothole detection, owing to their low cost and relatively simple detection algorithms. However, the accuracy of detection is lower than that achieved with other sensors such as cameras and lasers, because potholes are detected only when a vehicle’s wheels traverse a pothole. Moreover, false detections can occur with vehicles pass over manhole covers and speed bumps. Nevertheless, vibration-based pothole detection is advantageous given its low cost and simple methodology despite its limitations. Many studies have been performed in an effort to increase the accuracy of vibration-based detection by designing advanced algorithms and combining other sensor data [3,4,5]. Recently, smartphones have been proposed to support mobile sensing [6,7,8], but these methods have the same problems as vibration-based methods.

Laser scanning offers outstanding detection performance, compared to other methods. This approach is able to collect extremely detailed road-surface information using a technique that employs reflected laser pulses to create precise digital models [9,10]. Accurate 3D point clouds measure elevation in the surface, and this information is captured with the laser and then extracted by filtering the data for specific distress features by means of a grid-based processing approach. However, whereas laser scanning is highly precise, the equipment needed is expensive. Furthermore, this method cannot be applied over a wide area for fast pothole detection. 

Vision-based methods, however, are appropriate for accurately detecting potholes over a wide area at low cost. Many approaches using 2D images and video data have been studied. Pothole detection using 2D images was originally introduced by Koch and Brilakis [11]. Their method involved searching for specific pothole features and determining pothole regions. They used a remote-controlled robot vehicle prototype equipped with a webcam (an HP Elite Autofocus) installed at approximately 60 cm above the ground. Buza *et al.* introduced a new unsupervised vision-based method that does not require expensive equipment, additional filtering algorithms, or a training phase [12]. Jog *et al.* presented a new approach based on 2D recognition and 3D reconstruction for detecting and measuring the width, quantity, and depth of potholes using a monocular camera mounted on the rear of a vehicle [13]. 

Most existing vision-based pothole-detection algorithms use 2D images collected from the internet and specific high-resolution cameras. Moreover, these algorithms must be implemented on powerful desktop computers. Indeed, existing methods cannot support pothole detection using an embedded black-box camera. Thus, in this paper, we propose a pothole-detection system using a commercial black-box camera. We further propose a pothole-detection algorithm specifically designed for the black-box camera. Our pothole-detection system can be used as an efficient and low-cost pothole-maintenance system, and it can aid in the development of better road-maintenance policies. 

## 3. Proposed Pothole Maintenance System

**Figure 1:**
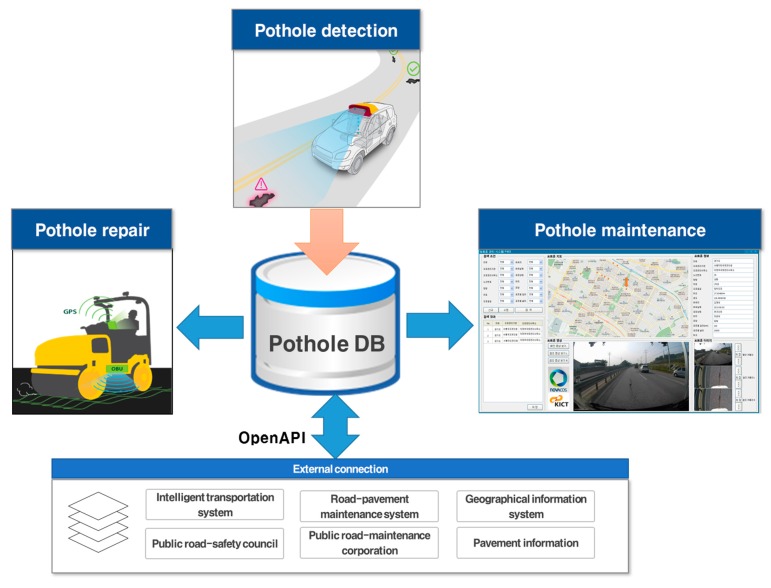
Proposed pothole-maintenance system.

Figure 1 depicts the proposed pothole-maintenance system with a pothole detector that uses a black-box camera. Pothole information, such as size, location and appearance, is collected by the pothole-detection system using the camera. The collected data is stored in the pothole database, and the pothole-maintenance server uses it for smart pothole maintenance. We developed new software for the pothole-maintenance server based on our previous pothole database system [14], as shown on the right in Figure 1. This software provides various pieces of information about potholes such as their video clips, images, regions, road authorities, route number of a road, driving direction, lane number of the road, type of road, latitude, longitude, collectors, collected date, type of pavement, location, shape, size, and comments. The pothole’s location is visualized on a digital map using the collected GPS data. Thus, users can easily see the distribution of potholes. Furthermore, the software accurately estimates the costs of pothole maintenance in the selected area. This way, transportation officials can easily and accurately develop road-maintenance policies and strategies with the software. Potholes can then be repaired smartly using the pothole-maintenance system such as our intelligent asphalt repair systems [15], and pothole information can be extended to other users and services via external connections and OpenAPI.

Insofar as the proposed pothole-detection system uses a single black-box camera, it is advantageous in terms of cost, utility, and scalability. A number of survey vehicles for pothole detection can be developed inexpensively, and data can be acquired quickly over a wide area. Road-maintenance agencies can accurately estimate the costs required to repair damaged road surfaces using the proposed pothole-maintenance system and the collected information regarding the location and size of potholes. In fact, the Korean government cannot accurately budget annual road-repair costs, because existing pothole-maintenance systems do not provide accurate pothole information.

## 4. Proposed Pothole-Detection Algorithm

**Figure 2:**
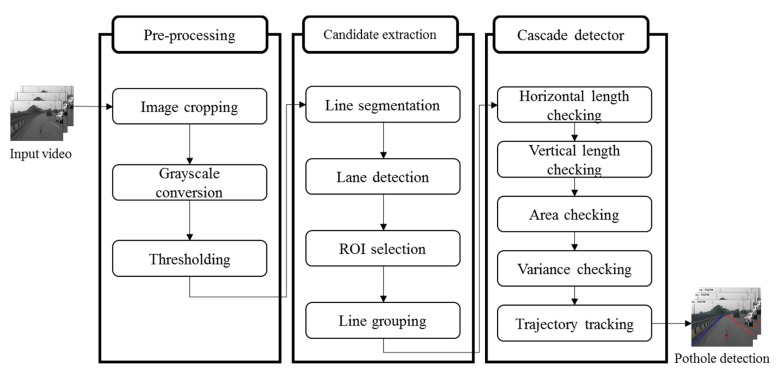
Proposed pothole-detection algorithm.

As shown in Figure 2, our proposed pothole-detection algorithm for the black-box camera is divided into three steps: pre-processing, candidate extraction, and cascade detection. First, we perform image cropping, grayscale conversion, and thresholding, to extract dark regions from the background. Next, we extract candidate pothole regions from these dark regions with four steps: line segmentation, lane detection, region-of-interest (ROI) selection, and line grouping. Finally, the algorithm determines whether candidate regions are potholes by inspecting various features, such as the length, area, variance, and trajectory. 

### 4.1. Pre-Processing

**Figure 3:**
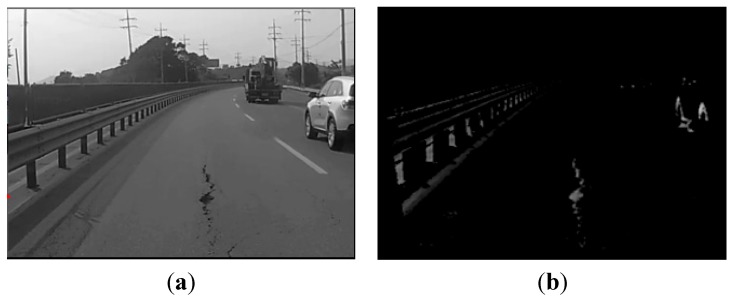
Pre-processing: (**a**) grayscale conversion; (**b**) thresholding.

The input images recorded by the black-box camera are cropped to a certain size in order to reduce the computational complexity. These resized images are converted to gray-scale, because potholes are generally devoid of color. Figure 3 shows the results after grayscale conversion and thresholding. Our proposal uses a histogram-based thresholding algorithm that transforms the grayscale image into a binary image. Either the maximum-entropy algorithm [16] or Otsu’s method [17] can be used for this transformation. In this study, we opted to use Otsu’s method, which separates the dark regions from the background as follows:

(1) $$B_{ij}=\{\begin{array}{c} \begin{array}{cc} 1, & if\;I_{ij}<T_{b} \end{array}\\ \begin{array}{cc} 0, & otherwise \end{array} \end{array}$$

where $B_{ij}$ is the pixel (i, j) in the binary image, $I_{ij}$ represents the corresponding pixel (i, j) in the input image, and $T_{b}$ is the threshold value for Otsu’s method. 

Otsu’s algorithm assumes that the image contains two classes ($C_{1}$ and $C_{2}$) of pixels, and the threshold value $T_{b}$ is determined by searching minimum intra-class variance (within-class variance) as follows:

(2) $$T_{b}=argmin_{0\leq x\leq L-1}\left\{H_{1}\sigma_{1}^{2}\left(x\right)+H_{2}\sigma_{2}^{2}\left(x\right)\right\}$$

where 

$\sigma_{1}^{2}\left(x\right)$ : the variance of class $C_{1}$ separated by a threshold x;

$\sigma_{2}^{2}\left(x\right)$ : the variance of class $C_{2}$ separated by a threshold x;

$H_{1}$ : the sum of pixels in class $C_{1}$;

$H_{2}$ : the sum of pixels in class $C_{2}$;

L : the maximum value of gray level.

As shown in Figure 3b, the pothole region in the middle of the image is segmented from the background. However, other regions, such as vehicle tires and the guard rails of a road, are also segmented from the background (we refer to such regions as *similar objects*). Thus, the pothole-detection algorithm removes similar objects in order to detect potholes accurately.

### 4.2. Candidate Extraction

**Figure 4:**
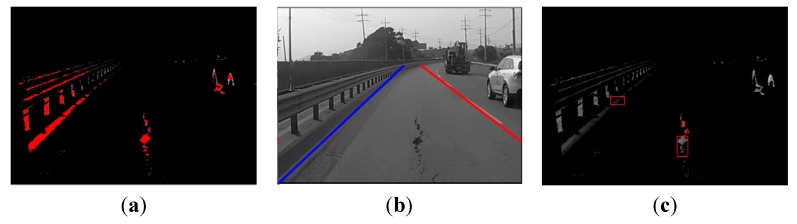
Candidate extraction: (**a**) line segmentation; (**b**) lane detection; (**c**) line grouping.

Following pre-processing, candidate extraction is performed to extract pothole candidate regions. This step consists of line segmentation, lane detection, ROI selection, and line grouping. Line segmentation involves investigating the line connectivity among pixels as follows:

(3) $$\left(S_{i,j},\;S_{i,j+1},\;\ldots ,\;S_{i,j+k}\right)=\{\begin{array}{cc} 1, & if\left(p_{i,j}\;AND\;p_{i,j+1}\;AND\ldots AND\;p_{i,j+k}\right)=1\\ 0, & elsewhere \end{array}$$

where $p_{i,j}$ is the pixel (i, j) in the binary image, $S_{i,j}$ represents the corresponding pixel (i, j) in the resulting image after line segmentation, and k denotes the threshold value for the horizontal length. Most noise generated as a result of thresholding during pre-processing step is removed with Equation (3), and lines longer than a certain size k remain. Figure 4a illustrates the results after line segmentation is applied to the image in Figure 3b. To render the candidate regions conspicuous, they are shown in red. 

As shown in Figure 4a, most similar objects, such as vehicle tires and guard rails are extracted with line segmentation. The lane is then detected in order to remove the remaining noise, as shown in Figure 4b. Only the road surface inside the two lane markings is used, and all of the values outside this area are removed. The algorithm for detecting lane proceeds as follows:

1. Brightness enhancement and thresholding;
2. Line scanning;
3. Selecting candidate points;
4. Detecting lanes.

**Figure 5:**
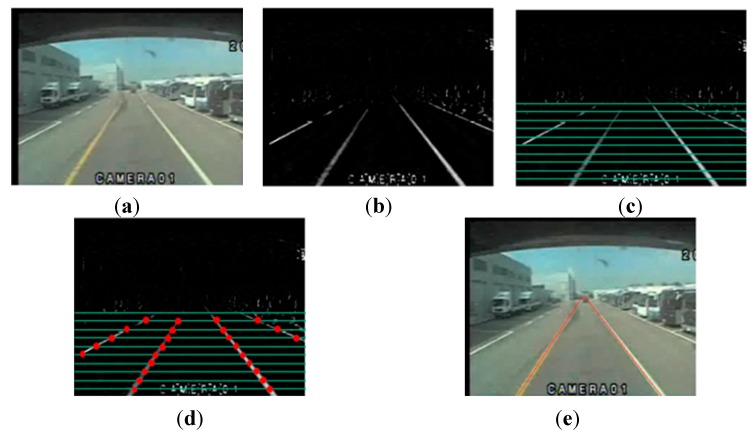
Lane detection: (**a**) input image; (**b**) brightness enhancement and thresholding; (**c**) line scanning; (**d**) selecting candidate points; (**e**) lane detection.

Typically, lanes markings on a road are brighter than the background. Thus, we can separate the lanes markings from the background by enhancing the brightness and by thresholding. Next, the candidate points for the lanes markings are selected by line scanning. Line scanning is performed before the vanishing point, because a lane cannot exist beyond the vanishing point. Finally, the selected lanes markings’ candidate points are connected by combining nearby points. Figure 5 provides examples for each step involved in lane detection. 

**Figure 6:**
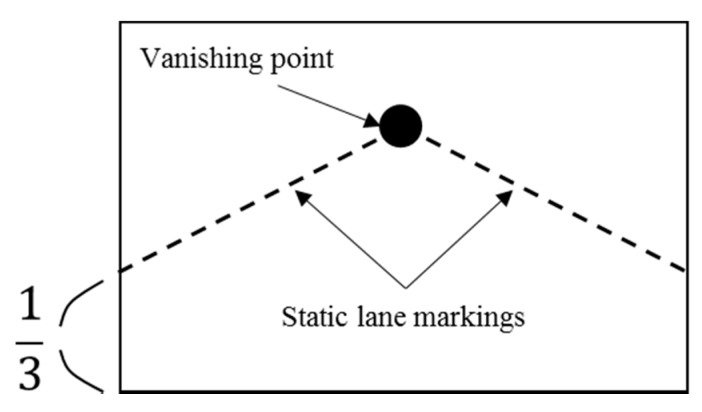
Static lane markings.

For roads without lane markings, and whenever lanes cannot be detected, we use a static lane-marking method. As illustrated in Figure 6, static lane markings can be generated using the vanishing point. Most lanes have two lane markings (one to the right of the vehicle, and one to the left). If one of the two lane markings is not detected, it is replaced by a static lane marking. That is, when a left lane marking is not detected, only that line is replaced by a static lane marking. 

**Figure 7:**
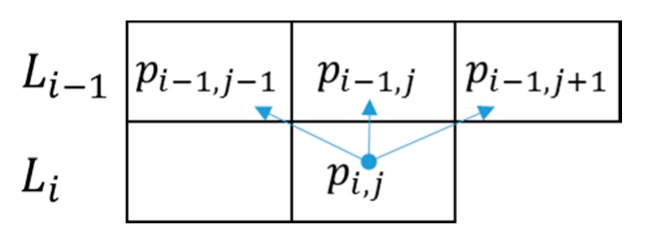
Line grouping.

The area between two detected lane markings is selected as the ROI. Line grouping is performed within the ROI, as illustrated in Figure 7. Let $L_{i}$ and $L_{i-1}$ denote two lines, where $L_{i}$ has a pixel value for pixel (i, j). If any of the pixels $p_{i-1,j-1}$, $p_{i-1,j}$, or $p_{i-1,j+1}$ has a value, the two lines, $L_{i}$ and $L_{i-1}$, can be considered to belong to the same group.

**Figure 8:**
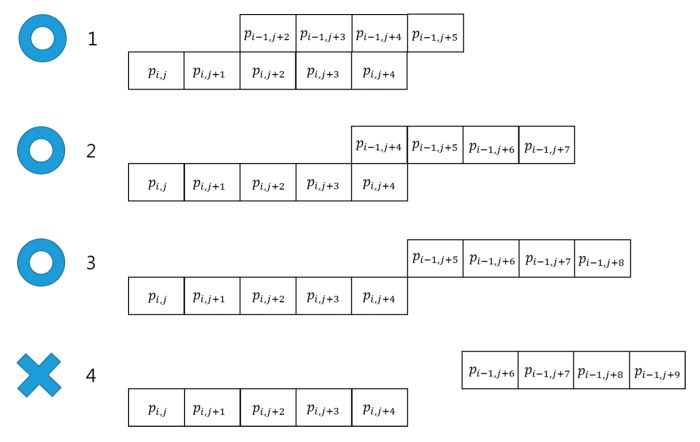
Examples of line grouping.

Figure 8 shows four line grouping examples. The two lines in the first, second, and third examples can be grouped together, but in the fourth example, the two lines do not belong to the same group. Line grouping also removes the noise created by thresholding, leaving only 2D regions, such as potholes, manholes, shade, road patches, *etc.*

Figure 4c shows four pothole candidate regions. Three of these candidate regions are not in fact potholes; only one region is actually a pothole. We can discriminate between true and false pothole candidates by using a cascade detector, as described in the next subsection.

### 4.3. Cascade Detector

After extracting candidate potholes, we employ a cascade-detection method in order to distinguish between true and false potholes:

(4) $$P=\{\begin{array}{cc} pothole\;region,\; & \begin{array}{c} if\;Maximum\;width>w\;AND\\ Maximum\;height>h\;AND\\ \begin{array}{c} Maximum\;Area>a\;AND\\ Maximum\;Variance>s\;AND\\ C_{y}\left(t\right)-C_{y}\left(t+1\right)<0 \end{array} \end{array}\\ non-pothole\;region,\; & elsewhere \end{array}$$

where w, h, a and s denote the threshold values for the width, height, area, and variance of the candidate regions, respectively, and where $C_{y}\left(t\right)$ denotes the y-coordinate for a candidate region in the video frame at time t. 

**Figure 9:**
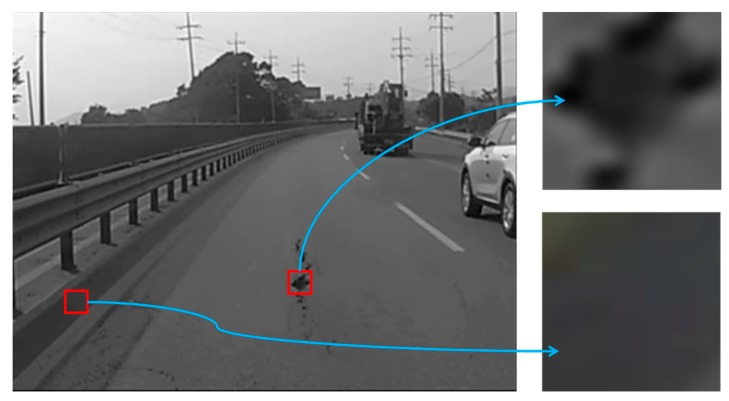
Difference between potholes and shade.

By filtering with the threshold values for the width, height, and area, we can remove minor cracks in the surface and other similar objects. A variance threshold is used to distinguish potholes from similar objects, such as road patches and shade. Figure 9 depicts the difference between a pothole and shaded region. Potholes have a coarse surface, whereas shaded regions have a flat surface. Thus, a pothole will have a high variance value, and a shaded region will have low variance. 

The final step for the cascade detector involves tracking the trajectory to remove the similar objects caused by other moving vehicles. When a vehicle moves into the ROI, the dark regions of the vehicle risk being detected as potholes. Normally, objects on the road’s surface (e.g., potholes) do not move. From the perspective of the black-box camera, however, they appear to move towards the camera. By contrast, vehicles traveling in the same lane move in the same direction as the camera. Thus, the proposed algorithm removes objects that are moving in the same direction as the camera.

**Figure 10:**
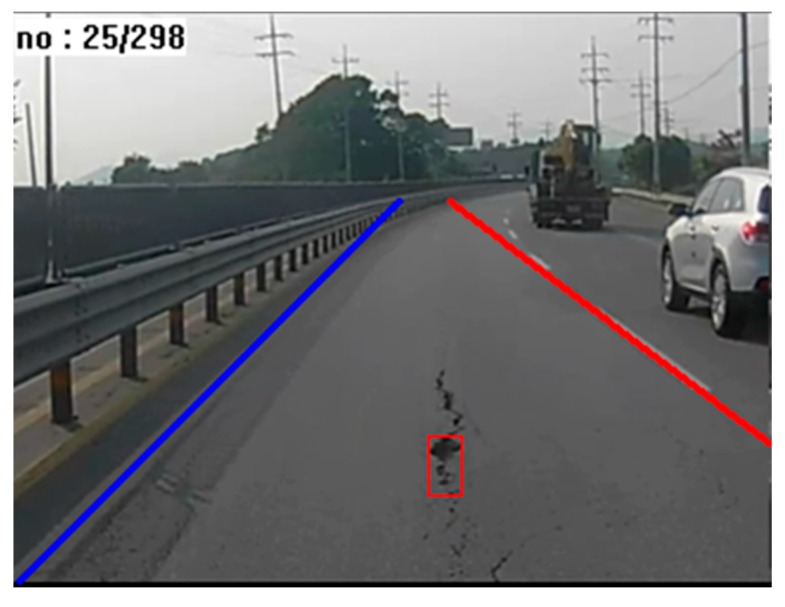
Result of pothole detection.

Figure 10 shows the final results from the pothole-detection system, representing the location and size of a pothole using a red box, and showing the two detected lane markings. The other three candidate regions shown in Figure 4c were removed by filtering for variance and size. 

## 5. Performance Evaluation 

### 5.1. Experimental Environment

**Figure 11:**
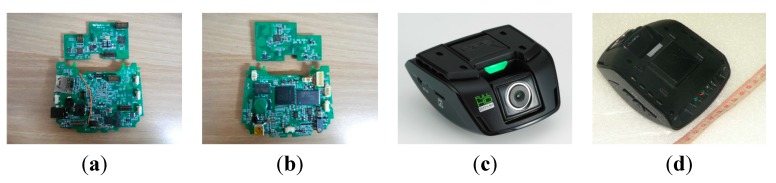
Black-box camera: (**a**) inside top view; (**b**) inside bottom view; (**c**) front view; (**d**) bottom view.

**Figure 12:**
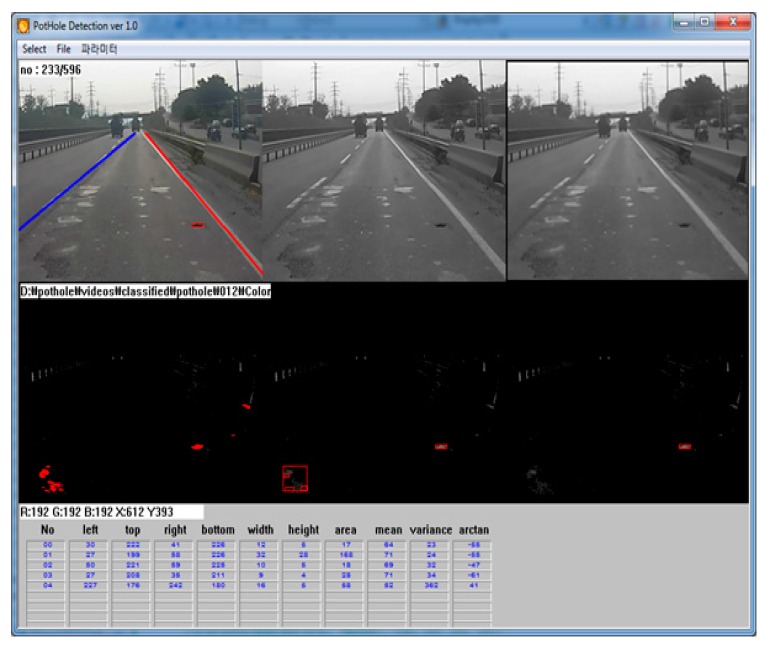
Simulation software for pothole detection.

In this paper, we collected pothole video data through recordings made with a black-box camera mounted on the front windshield of a survey vehicle (typically, a black-box camera is installed on the front windshield), as shown in Figure 11. The resolution and frame-rate were 1920 × 1080 pixels and 27 f/s, respectively. The camera lens could be tilted by the user. The recorded video data was saved in H.264 format with 32 GB of memory. A Cortex-A8 application microprocessor was used for recording and processing the video data. Before implementing the proposed pothole-detection algorithm in the black-box camera, we developed simulation software using C++, as shown in Figure 12. This software displays the pothole-detection results and also the parameters such as the width, height, and variance. 

Table 1 details the values of the parameters used in this study. Each value depends on the size and shape of a particular pothole. With the exception of $T_{b}$(the threshold for Otsu’s method), all values were set empirically such that they were the most suitable for distinguishing potholes from similar objects.

During pre-processing, the images were cropped from (560, 240) to (1200, 720) to generate images that were 640 × 480 pixels in size. The cropping size was decided based on the processor performance. If we use more powerful processors, we can use larger size of images.

**Table 1:** Parameters used in this study.

Parameter	Value
$T_{b}$	This value depends on the input image
k	4
w	15
h	4
a	w×h
s	100

### 5.2. Results

A total of 20 video clips of potholed roads were collected using our black-box camera along National Highway 6 in May 2015. The video was recorded in sunny weather at noon. In this study, we do not consider conditions at night or weather conditions such as rain, snow, or wind. 

**Figure 13:**
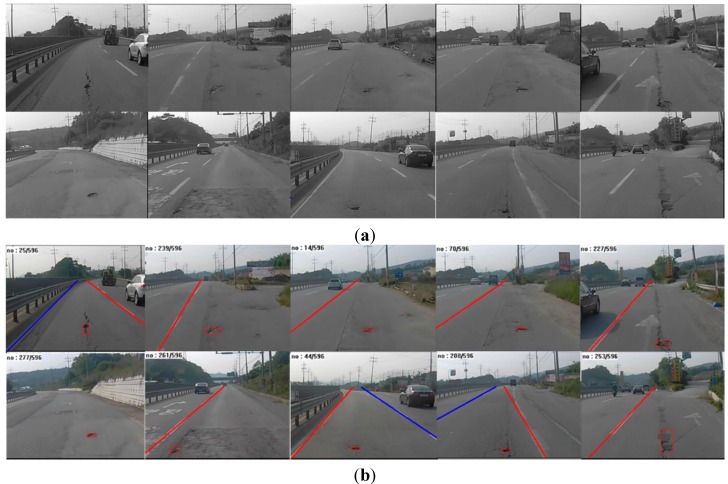
Examples of pothole detection: (**a**) input images; (**b**) results of pothole detection.

Figure 13 shows examples of original input images and their corresponding pothole-detection results. As shown in Figure 13b, lane markings were not always detected on both the left and the right, but the algorithm nonetheless correctly detected potholes by using the static-lane method. Moreover, potholes could be located even in cases where no lane markings were detected. This means that our algorithm can accurately detect potholes on various road surfaces and lane conditions. 

**Figure 14:**
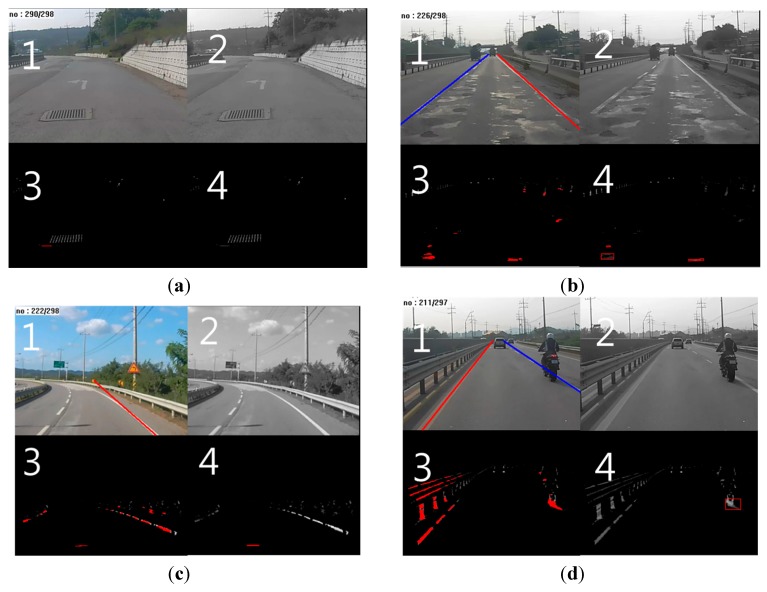
Similar object removal: (**a**) manhole; (**b**) patchy surface; (**c**) shaded regions; (**d**) moving vehicle.

Figure 14 shows examples of similar objects removed by the proposed algorithm. Images numbered 1, 2, 3, and 4 are the results after pothole detection, grayscale conversion, line segmentation, and line grouping, respectively. 

Figure 14a shows a manhole that was removed with line segmentation. No segmented lines were found in the region of the manhole in Image 3. The manhole’s surface has a constant grid pattern. Thus, its pixels were not connected to each other. Figure 14b shows a patchy surface and its removal through variance filtering. We can see grouped lines on the patchy regions in Image 4. However, the surface area corresponding to these patchy regions was not sufficiently distinct to result in a high degree of variance. 

Figure 14c shows a shady area and its removal with variance filtering. Figure 14d shows a moving vehicle that was removed via trajectory tracking. Line grouping was conducted on the rear tire of the motorcycle in Image 4. All conditions related to size and variance were satisfied in this case. Yet, the tire was removed because it moved in the same direction as the black-box camera. In order to determine the accuracy of the proposed method, we manually counted the number of true positives, false positives, and false negatives. We did not count true negatives, because doing so with continuous video data is considerably ambiguous and uncertain:

- True Positives (TPs): correctly detected as a pothole;
- False Positives (FPs): wrongly detected as a pothole;
- False Negatives (FNs): wrongly detected as a non-pothole

Table 2 shows the performance results for the proposed algorithm according to the number of TPs, FPs, FNs, and its sensitivity and precision. Sensitivity refers to the ratio of correctly detected potholes to actual potholes, and precision refers to the ratio of correctly detected potholes to the total number of detected potholes. The sensitivity and precision are thus calculated as follows:

- Sensitivity (True-positive rate): TP/(TP + FN);
- Precision (Positive-predictive value): TP/(TP + FP).

As shown in Table 2, the proposed method resulted in an overall sensitivity of 71%, with 88% precision. Thus, the method is more precise than it is sensitive. This means that the algorithm is robust to various kinds of similar objects such as manholes, patches, shade, and moving vehicles. There were fewer FPs than FNs, owing to the diversity of the shape and size of potholes. Moreover, the proposed algorithm wrongly detected potholes that were especially bright or flat. We think that the algorithm can be improved by adding more conditions to the cascade detector with only a minimal increase to the algorithm’s complexity. Thus, we confirmed that the proposed pothole-detection system using a black-box camera has can be utilized to collect pothole information. In addition, we expect that our black-box camera with the proposed algorithm can be used successfully as a pothole-alert system, owing to its robustness to similar objects. 

**Table 2:** Performance results.

Performance	Value
TP	22
FP	3
FN	9
Sensitivity (True-positive rate)	71%
Precision (Positive predictive value)	88%

## 6. Conclusions

In this paper, we propose a novel pothole-detection system using a black-box camera, and we further design a unique pothole-detection algorithm. The algorithm was simulated on a desktop computer, and then embedded in a black-box camera. 

The proposed pothole-detection algorithm was designed and implemented in consideration of the limited computing power of the embedded systems in black-box cameras. The experimental results demonstrate that the proposed algorithm can correctly remove various types of similar objects, such as patches, manholes, shade, and moving vehicles. By doing so, pothole regions can be detected correctly. The overall sensitivity and precision reached 71% and 88%, respectively. Thus, the algorithm is considerably robust to similar objects. In some cases, however, the proposed system failed to detect potholes that were especially bright or flat. 

We tested the proposed algorithm in sunny weather. Consequently, a comprehensive evaluation is needed under various weather conditions in future research. Moreover, we shall collect more pothole video data during subsequent evaluations. We believe that the evaluation results in this paper are insufficient for demonstrating a normalized performance. The algorithm for detecting lane markings was simply designed, owing to the limited computing environment of the black-box camera. When the intensity of sunlight quickly changed, false detections occurred. We intend to improve this algorithm with only minimal increases to the complexity of the overall system. Similar object removal with trajectory tracking also deserves improvement, because similar objects caused by vehicles in front of the pothole detector was sometimes detected as a pothole. 

Thus, multiple issues remain with the proposed pothole-detection algorithm. Nevertheless, we confirmed that a pothole-detection system using a typical black-box camera has the potential for use as an automatic and smart pothole-maintenance system. 

We designed the pothole-maintenance system based on various opinions collected from the experts of pavement management system in Korea Institute of Civil Engineering and Building Technology. We will apply the pothole-maintenance system in collaboration with the road authorities of Seoul city in Korea.
